# Entrepreneurial Intention and Delayed Job Satisfaction From the Perspective of Emotional Interaction: The Mediating of Psychological Capital

**DOI:** 10.3389/fpsyg.2022.925460

**Published:** 2022-06-14

**Authors:** Boxiang Na, Noor Hazlina Ahmad, Chenxiao Zhang, Yan Han

**Affiliations:** ^1^School of Management, Universiti Sains Malaysia, Penang, Malaysia; ^2^School of Architecture and Artistic Design, University of Science and Technology Liaoning, Liaoning, China; ^3^School of Humanities and Social Science, Beijing Institute of Technology, Beijing, China

**Keywords:** emotional interaction, entrepreneurial intention, psychological capital, occupational delay of gratification, mediating effect

## Abstract

The coronavirus disease 2019 (COVID-19) pandemic has exacerbated the labour shortage, and promoting entrepreneurship to spur job creation is one of the most effective strategies to address this problem. Entrepreneurs must lengthen their employment or start-up cycles due to COVID-19 normalisation. Consequently, the impact of career willingness to delay satisfaction on entrepreneurial ambition is investigated in this research *via* an online survey in Jiangsu Province, China. The findings show that students with a high level of career delayed contentment has a higher level of entrepreneurial intention (EI), implying that career delayed contentment intention influences EI positively. Psychological capital (PC) modifies this process, increasing the influence of job delayed satisfaction on EI by strengthening PC. PC’s significant components are self-efficacy, hope, optimism, and flexibility. This study combines the willingness to wait for satisfaction with the willingness to start a business, providing a valuable reference for reducing the work condition caused by the COVID-19 epidemic.

## Introduction

The negative economic impact of coronavirus disease 2019 (COVID-19) resulted in a significant increase in unemployment and a decrease in job seekers’ willingness to look for work at the beginning of 2020 (Mitman and Rabinovich, 2021; Sjoquist and Wheeler, 2021). As a result, people are more likely to start their businesses or further their education (Chodorow-Reich and Coglianese, 2021). During the epidemic, a new area of entrepreneurship research emerged: researching the factors that support students’ career development and future entrepreneurial decisions (Verma and Gustafsson, 2020; Saleh Al-Omoush et al., 2021).

The relationship between entrepreneurial intention (EI) and psychological capital (PC) focuses on those factors influencing EI (Haddoud et al., 2022; Laouiti et al., 2022). These studies concentrate on the positive effects of PC, specifically self-efficacy, optimism, hopelessness, and adaptability, the four components of PC (Douglas et al., 2021). It is a research perspective to explore the role of psychology based on the interaction between EI and entrepreneurial attitude (EA) (Pérez-Pérez et al., 2021; Uysal et al., 2021). EA indirectly guides entrepreneurial behaviour through one’s intentions (Gieure et al., 2020). It has been discovered that there is a strong link between high PC and EI among college students (Hunt et al., 2022; Núñez-Canal et al., 2022). Delay in career satisfaction can also increase a person’s willingness to persevere and, as a result, start a business (Zhao et al., 2021). The greater the one’s ability to postpone gratification, the greater the one’s PC, and the greater the one’s chances of achieving long-term goals and acquiring more adequate resources (Aschheim et al., 1974). Students who have a high level of career postponement satisfaction, in contrast, may have high expectations for dealing with job stress (Jaensch et al., 2015). Existing research also indicates that PC plays a critical mediating role in the relationship between job stress and career delay satisfaction among college students (Sun et al., 2021).

Previous studies on students’ EI focused on external factors such as positive psychological fields rather than students’ career satisfaction delays (Badri and Hachicha, 2019; Morrish and Jones, 2020). Delayed career satisfaction significantly impacts employers’ current work attitudes and students’ career choices (Jenson et al., 2020). In fact, no comprehensive research on students’ delayed career satisfaction has been conducted. Although nurses have received the most attention in delayed gratification research, the business profession is also worth investigating. According to some studies of EI, entrepreneurship education for students will cultivate students’ EI. What aspects of entrepreneurship education should be taken into account? Is this still true among students at the International Business School of Suzhou of Xi’an Jiaotong-Liverpool University (IBSS) in Suzhou, Jiangsu Province, China? Furthermore, PC plays a vital role in bridging the gap between college students’ employment and career satisfaction. Is there a factor that takes delayed gratification into account when determining EI? What is the link between these three variables? By conducting the research, the role of the emotion variable is defined to affect the process of students starting up a new business. Then, a new teaching model is expected to explore to cultivate students’ self-awareness of innovation and entrepreneurship. A college education is different from the teaching model of high school teaching activity, indicating that internal factors, such as personal traits, and external factors such as the economic situation of the original family, influence students’ decision-making besides diathesis cultivation (Cavalcante et al., 2021). As a result, this study will quantitatively investigate the mediating effect of career delayed gratification on EI and PC using a questionnaire survey of IBSS students to verify the new stimulation factor of EI.

## Literature Review and Hypothesis Development

In studies focusing on EA, individuals’ attitudes toward entrepreneurship are initially positive or negative feelings (Munawaroh, 2020). Theory of plan behavior (TPB) is widely regarded as an appropriate tool for analysing the relationship between EI and EA (Gough, 2019), and TPB can directly and effectively support the impact of EI. According to this theory, EA is an indirect act of entrepreneurship based on one’s intentions. Subjective norms and perceived behavioural control also influence entrepreneurial behaviour. Obviously, EA and EI are inextricably linked. In other words, EA and EI precede one another, and cultivating a positive attitude is the most crucial factor in influencing students to become entrepreneurs in the future (Sharahiley, 2020). Although this study reveals the importance of subjective norms on students’ EI, it also confirms that cultural constraints limit students’ cognitive abilities. Those from Western cultural backgrounds appear to have fared better. Consequently, the mediation EA elements are guaranteed to be the common factors that drive EI.

### The Influence of Psychological Capital on the Formation of Entrepreneurial Intention

It is proposed that PC persuades people to set goals, envisions the future, and achieves outcomes (Li et al., 2021). Therefore, students with high PC are more interested in EI, which is defined by a focus on life’s strengths and positivity in order to assist ordinary people in discovering useful, acceptable, and developmental phenomena (Esfandiar et al., 2019; Meoli et al., 2020; Hoang et al., 2022). Positive organisational behaviour is viewed as a factor that can be measured, developed, and managed effectively in order to improve performance (Luthans et al., 2006). PC components include self-efficacy, optimism, hope, and resilience (Ye et al., 2020). PC, a medium that indirectly regulates people’s attitudes and emotions, has previously focused on EA-related research. PC improves the impact of students’ EA on EI (Mahfud et al., 2020). According to studies, EA orientation, PC, and social capital have synergistic and interactive effects on EI. People with a strong sense of self-efficacy, optimism, hope, and resilience use motivation, cognition, and action to achieve their objectives (Mahfud et al., 2020). To wrap up, we proposed the following research hypothesis 1:

***Hypothesis 1: PC has an optimistic prediction of students’ EI directly.***

### Occupational Delayed Gratification and Psychological Capital

The ability to resist activities such as relaxation and entertainment that are not conducive to continued work is referred to as delayed occupational gratification (Sun et al., 2021). Individuals with a high level of delayed job satisfaction may achieve task goals in the future through delayed rewards (Stamolampros et al., 2019). Pressure contributes to the depletion of PC, and as pressure increases, entrepreneurs will prefer impulsive behaviour to delayed gratification (Min et al., 2015). Individual career decisions and enterprise performance in their current employment status are influenced by the amount of time it takes to receive satisfaction (Martínez-León et al., 2018; Zhao et al., 2021). A high level of delayed job satisfaction can boost PC’s self-efficacy, which in turn influences EI (Stratman and Youssef-Morgan, 2019). When people meet their interest choices, delayed gratification encourages them to forego short-term instant gratification in favour of more valuable long-term career goals, which is beneficial to students’ career development (Xu, 2021). Professional delayed satisfaction refers to an individual who is willing to forego rest, entertainment, or impulsive behaviour that is not conducive to current job opportunities. In exchange, they pay more attention to the instant gratification ability of self-regulation in order to better complete work tasks, earn more profit, achieve higher career goals, and have a series of more valuable long-term results. Based on the above analysis, we proposed the following hypotheses 2a and 2b:

***Hypothesis 2a: Students’ occupational delay of gratification can impinge on their PC positively.***

***Hypothesis 2b: Occupational delay of gratification may negatively predict students’ EI.***

### The Mechanism of Psychological Capital

Hope is a PC component that combines a sense of accomplishment with goal-oriented energy and plans to achieve those goals (Peterson et al., 2008). As a component of PC, hope can influence an individual’s EI through two dimensions, namely, willpower and road power (Ephrem et al., 2019). Hope can help entrepreneurs set high-level goals and convince them of their ability to capitalise on business opportunities (Jayanti and Raghunath, 2018). If hope and self-efficacy are positive motivators, then resilience is a coping mechanism in difficult situations, and resilience is a dimension of PC (Han et al., 2022). The ability to replicate and adapt in the face of loss, hardship, or adversity is defined as resilience (Yuan et al., 2022), and it has an impact on entrepreneurial outcomes. As a component of PC, optimism cannot be directly classified as a mediator because it can, in some cases, negatively impact EI (Mahfud et al., 2020). Excess optimism and positivity can lead to risky decisions. Consequently, a healthy dose of optimism can assist businesses in succeeding (Bonaparte et al., 2017; Bruhin et al., 2018). Based on the entrepreneurial process, self-efficacy is defined as the belief that an individual can successfully perform the role and task of an entrepreneur (Chen et al., 2016). People with a strong sense of self-efficacy are more likely to exert tremendous effort and perseverance, resulting in a higher level of self-efficacy (Hsu et al., 2014). Successful experiences can increase efficacy, while unsuccessful ones can decrease it (Bandura, 1978; Luthans et al., 2006). As a result, improving PC skills such as self-efficacy, hope, optimism, and resilience is a powerful way to prepare students for future entrepreneurship (Ephrem et al., 2019). Therefore, we proposed the following hypothesis 3:

***Hypothesis 3: The PC of college students mediates the relationship between career delayed gratification and EI.***

## Methodology

According to the previous research mentioned above, researchers have validated the relationship between EI and PC and the relationship between the occupational delay of gratification. However, the relationship between EI and the occupational delay of gratification was not ensured. This research was expected to clarify the relationship between those three variables based on the samples of students in IBSS. As a result, quantitative research was a suitable method for demonstrating the original hypothesis and constructing the relationship between EI and occupational delay of gratification. Compared with quantitative research, qualitative research was more helpful in proposing the hypothesis but not persuasive to readers. Based on the research method of other researchers, such as Liñán (2009) and Görgens-Ekermans and Herbert (2013), a quantitative method was used in this research.

### The Questionnaire

This study investigates EI, PC, and delayed career satisfaction by online survey using Wenjuanxing, a platform sharing questionnaires to target volunteers. The Individual Entrepreneurial Orientation Scale is used to determine whether or not the sample has a high level of EI (Santos et al., 2020). A 7-point Likert Scale with six questions was used to assess EI (Liñán and Chen, 2009; Jena, 2020). PC is thought to play an essential role in regulating students’ EI. This study employs a questionnaire survey to assess the effectiveness of PC based on several factors. There are, however, some drawbacks to using a single questionnaire. Other factors, such as self-efficacy and hope, must be considered. In addition, EI interacts with all elements. Finally, we discovered that Görgens-Ekermans and Herbert (2013) used the six-item PQS-24 scale to assess PC. The scale evaluates PC holistically, allowing it to predict the role of intermediaries better. Ray and Najman (1986) used the Q-classification technique to develop the Delay of Gratification Questionnaire (DGQ) to investigate people’s proclivity to delay gratification in general. DGQ has some shortcomings as a scale measuring students’ vocational delay of gratification, despite being detailed and appropriate for students with no work experience and uncertain future career plans. One of them is a less professional relationship. While the questionnaire can infer career delayed satisfaction orientation, it is not a reliable and direct description, and social expectations may influence DGQ results. Another widely used measurement tool is the Multidimensional Work Ethic Scale (MWEP). It was proposed by Miller (2001) as part of a work ethics study, and it treats gratification delay as a separate dimension. The main disadvantage of the MWEP method is that it is impossible to measure the willingness of delayed satisfaction in occupation as a single dimension. From the perspective of measuring students’ (EI), Hernandez-Sanchez (2020) assesses EI using the Entrepreneur Orientation Questionnaire (EOQ), a six-item scale with high validity.

The PQS-24 questionnaire is superior in terms of PC, but too many items and PC elements are complicated. As a result, eight items were chosen to comprise the PC section of the questionnaire (2 items per element). The original reverse scoring item was changed into an affirmative sentence to simplify the research process and ensure that all interviewees understood the project. To maintain the benefits of the methods mentioned earlier, adjustments are made according to DGQ and MWEP procedures. When analysing EI, the conceptual framework proposed by Liñán (2009) is used. As a statistical method, the EI section of the questionnaire is extracted using the 5-point Likert subscale. Some basic information questions were cited before the questionnaire to obtain accurate goals, including three questions about entrepreneurial attempts, as well as the gender and age of the interviewer. As a result, the questionnaire is divided into four sections, totalling 34 questions (Appendix). The parallel study conducted three tests in different groups to improve the measurement’s reliability. To ensure that items were reduced in reliability and eliminated, each group was required to complete and distribute questionnaires as well as calculate data. The questionnaire was finally designed and shared with volunteer students after three iterations. The problems impacted by external factors are listed above for analysis. The experiment was carried out using the SPSS Process.

### Participants

A total of 380 mainland Chinese students were selected among Junior and Senior undergraduate students as well as postgraduate students in IBSS to collect enough samples to ensure the relationship among the three variables, who were not classified by definite grades but by degree. Then, 340 students were selected as the final samples (135 students were undergraduate students and 205 students were postgraduate students). Furthermore, the response rate of participants is 89.47% (340/380 = 89.5%). From the perspective of knowledge, most students studied Management, Project Management, and Business Administration (approximately 91.18%). The remaining 8.82% of students studied Financial Mathematics and Finance and Accounting. Of the initial cohort of 340 students, 227 were women, and 113 men, accounting for approximately 66.76 and 33.24% individually. The first three basic questions identified eligible women and men who matched the selection criteria: Do you already have your own business? Does someone in your family have their own business? Do any of your friends have their own businesses? Students with one or more than one positive answer to those questions are the objectives we need to conduct the following test. The age range is between 23 and 31, with an average of 24.47 (standard deviation = 1.362). There were 77 students aged 23 years. Moreover, the students aged 24 years were 101. The central part of volunteers in the research was the students aged 25 years with 142 samples. Notably, students aged 28 years and 31 years took a small account (only 15 and 5 samples individually) (Table 1).

**TABLE 1:** The descriptive statistics of samples.

	Frequency	Percent	Valid percent	Cumulative percent
Do you already have your own business?	Yes	340.00	100.00	100.00	100.00
Does someone in your family have their own business?	Yes	340.00	100.00	100.00	100.00
Do any of your friends have their own business?	Yes	340.00	100.00	100.00	100.00
Age	23	77.00	22.65	22.65	22.65
	24	101.00	29.71	29.71	52.35
	25	142.00	41.76	41.76	94.12
	28	15.00	4.41	4.41	98.53
	31	5.00	1.47	1.47	100.00
	Total	340.00	100.00	100.00	
Gender	Male	113.00	33.24	33.24	33.24
	Female	227.00	66.76	66.76	100.00
	Total	340.00	100.00	100.00	

With the permission of the Chair of the Research Ethics Committee of Xi’an Jiaotong-Liverpool University (RESC), questionnaires were designed and distributed to students. All the volunteer students who took part in the project were informed that all of their personal information and details would be used in the research in order to keep their lives as normal as possible. To ensure that all participants understood the research’s purpose and risks, they were required to sign a consent form. All the questions posed had to be answered anonymously and confidentially, and they had to adhere to the American Psychological Association (APA) ethical standards. Due to the COVID-19 pandemic and a large number of samples, almost all the research and data collection took place online. The questionnaire shown to Management students was answered *via* the shared QR code, and students studying Financial Mathematics, Finance, and Accounting were required to answer the questionnaire *via* paper material. All the results were collected and screened by Excel after eliminating unsuitable participants.

## Results

### The Descriptive Statistics of Samples

The frequency analysis shows that the distribution of “Do you have your own business?” was dominated by the “yes” answer, accounting for 100.00%. The distribution of “Do your family members have their own business?” was mainly “yes,” accounting for 100.00%. The distribution of “Do your friends have their own business?” was mainly “yes,” accounting for 100.00%. Therefore, the samples selected according to this question were suitable. The dominant age of samples was “25,” accounting for 41.76%. Additionally, the distribution of gender samples was mainly “female,” accounting for 66.76%.

### Confirmatory Factor Analysis of Questionnaire

All the data collected from the questionnaire were processed and analysed. According to the data process, different items in various classifications were all in a good situation. For the EI part, the total Cronbach’s alpha of the 9 items was 0.901, and the value of Cronbach’s alpha if Item Deleted was 0.890, 0.889, 0.894, 0.892, 0.889, 0.887, 0.891, 0.892, and 0.890 for each item, which was all lower than 0.901. Therefore, all the part items are necessary. For the self-efficacy part, the total value of Cronbach’s alpha was 0.869, higher than all the items’ value of Cronbach’s alpha if the item was Deleted. Consequently, all the items in the self-efficacy part were also significant. For the hope part, the result was 0.879, higher than each result of Cronbach’s alpha value if items were deleted, which means that all the items should be adapted. When discussing the resilience part, each item’s value of Cronbach’s alpha was lower than 0.867 if items were deleted. When discussing the optimism part, each item’s value of Cronbach’s alpha was also lower than the whole part. The same situation was also suitable for vocational delay of gratification; all the results of Cronbach’s alpha if items deleted were lower than the total value. Obviously, all the items in each part were valuable.

In a word, the results showed that the overall Cronbach’s alpha coefficient of the scale was more significant than 0.7, and the Cronbach’s alpha coefficient values corresponding to the six dimensions were greater than 0.7, indicating that the internal consistency of the questionnaire was good. As a result, the confirmatory factor analysis of the survey results was excellent (Gerber, 2005).

Total correlation calculation aimed to identify the correlation between each item and the overall score. Items whose correlation coefficient between item score and the overall score was lower than 0.3 were supposed to be deleted (Gerber, 2005). After the confirmatory factor analysis, no items were expected to be deleted as the correlation between items and the overall score in this questionnaire was higher than 0.3. The details are shown in Table 2. The results showed that most items were related to the entirety and had discrimination.

**TABLE 2:** Reliability analysis of the questionnaire.

	Cronbach’s Alpha	Total Cronbach’s Alpha
EI	0.901	0.926
Self-efficacy	0.869	
Hope	0.879	
Resilience	0.867	
Optimism	0.873	
Vocational delay of gratification	0.877	

### Validity Analysis of the Questionnaire

According to the test results in the analysis Table 3, the Kaiser–Meyer–Olkin (KMO) value of this study was 0.919, significantly greater than 0.7. In Bartley’s spherical test, the approximate chi-square value was 6,211.449, and the probability of significance was 0.000, less than 0.01. Therefore, the rejection of the null hypothesis of Bartley’s sphericity test indicated that the validity structure of the questionnaire was good, which could be used for factor analysis (Gerber, 2005). At the same time, the common degree table of each index was obtained as in Table 3.

**TABLE 3:** Kaiser–Meyer–Olkin (KMO) and Bartlett’s test.

Kaiser–Meyer–Olkin measure of sampling adequacy	0.919
Bartlett’s Test of Sphericity	Approx. Chi-square	6211.449
	df	561
	Sig.	0.000

According to the analysis of the results in Table 4, the common degree of each index was greater than 0.40, which indicated that the influence of each index on the scale was significant and should be retained. The total variance analysis was carried out on this basis, and Table 5 was obtained.

**TABLE 4:** Common metre of each indicator.

	Initial	Extraction
A1 Being an entrepreneur implies more advantages than disadvantages to me.	1.000	0.576
A2 A career as an entrepreneur is attractive to me.	1.000	0.592
A3 If I had the opportunity and resources, I would like to start a firm.	1.000	0.515
A4 Being an entrepreneur would entail great satisfaction for me.	1.000	0.544
A5 Among various options, I would rather be an entrepreneur.	1.000	0.591
A6 I am ready to do anything to be an entrepreneur.	1.000	0.654
A7 My life goal is to become an entrepreneur.	1.000	0.550
A8 I will make every effort to start and run my own firm.	1.000	0.543
A9 I have the intention to start a firm someday.	1.000	0.576
B1 I believe that I can analyse long-term problems and find solutions.	1.000	0.613
B2 Within the scope of my work, I believe I can help set goals/objectives.	1.000	0.657
B3 I believe that I have contributed to the discussion of teamwork strategies.	1.000	0.791
B4 I believe that I can contact people outside of the organisation (such as suppliers, customers, merchants) and discuss them with them.	1.000	0.640
B5 I believe that I can present information to a group of colleagues.	1.000	0.618
C1 If I am involved in troubles at work, I can think of many ways to get rid of it.	1.000	0.673
C2 At present, I am full of energy to complete my work goals.	1.000	0.686
C3 At present, I think I am quite successful in my work.	1.000	0.565
C4 At present, I am achieving the goals I set for myself.	1.000	0.819
C5 I can find out many ways to achieve my current work goals.	1.000	0.675
D1 It’s easy for me to recover from setbacks at work and move on.	1.000	0.617
D2 I usually cope with pressure in my work easily.	1.000	0.641
D3 I can solve most of the problems in my work.	1.000	0.596
D4 I can complete the tasks by myself if they must be done.	1.000	0.809
D5 In my current job, I reckon that I can handle many things simultaneously.	1.000	0.674
G1 In my work, I usually expect the best results when encountering uncertain things.	1.000	0.604
G2 I always find out the positive side of things when I work.	1.000	0.662
G3 I am optimistic about what will happen in the future of my work.	1.000	0.805
G4 I always believe that “There will be a rainbow after every storm, so don’t be pessimistic.”	1.000	0.667
G5 If something goes wrong, if I work wisely, it can be changed.	1.000	0.647
H1 If I want to buy something, I always wait until I can afford it.	1.000	0.700
H2 Completing a tough job can show your ability.	1.000	0.644
H3 s a middle-level manager of a company, you prefer to be the leader of a team that is not very good temporarily, but with great potentiality.	1.000	0.656
H4 You will do all in your full strength to look for all chances to get the big client though there are many unexpected difficulties in the process.	1.000	0.626
H5 If your work is so stressful that you are uncomfortable to bearing with it, you will look for ways to relieve pressure and then continue to do a good job.	1.000	0.794

**TABLE 5:** Total variance explained.

Component	Initial eigenvalues			Extraction sums of squared loadings			Rotation sums of squared loadings		
	Total	Variance (%)	Cumulative (%)	Total	Variance (%)	Cumulative (%)	Total	Variance (%)	Cumulative (%)
1	10.110	29.736	29.736	10.110	29.736	29.736	5.167	15.196	15.196
2	3.012	8.860	38.596	3.012	8.860	38.596	3.478	10.229	25.425
3	2.749	8.086	46.682	2.749	8.086	46.682	3.378	9.936	35.361
4	2.213	6.509	53.191	2.213	6.509	53.191	3.375	9.926	45.287
5	2.099	6.174	59.365	2.099	6.174	59.365	3.327	9.786	55.073
6	1.836	5.401	64.767	1.836	5.401	64.767	3.296	9.693	64.767
7	0.723	2.127	66.894						
8	0.709	2.085	68.979						
9	0.659	1.938	70.917						
10	0.622	1.828	72.745						
11	0.591	1.738	74.483						
12	0.555	1.632	76.115						
13	0.541	1.590	77.705						
14	0.535	1.573	79.278						
15	0.522	1.536	80.815						
16	0.488	1.436	82.251						
17	0.469	1.380	83.631						
18	0.458	1.346	84.977						
19	0.432	1.272	86.249						
20	0.425	1.249	87.498						
21	0.403	1.186	88.684						
22	0.398	1.169	89.853						
23	0.372	1.093	90.946						
24	0.368	1.083	92.030						
25	0.354	1.042	93.072						
26	0.324	0.953	94.025						
27	0.311	0.914	94.939						
28	0.304	0.895	95.834						
29	0.289	0.851	96.685						
30	0.257	0.756	97.441						
31	0.246	0.723	98.164						
32	0.226	0.664	98.827						
33	0.208	0.613	99.440						
34	0.190	0.560	100.000						

It can be seen that there were six common factors with larger eigenvalues. The factor analysis results also showed that the explanation rate of the total variance of the factors was 64.767%, which was more than 60%. Therefore, it could be ensured that the designed scale has a better explanation (Gerber, 2005). Then, we obtained the scree plot.

**FIGURE 1:**
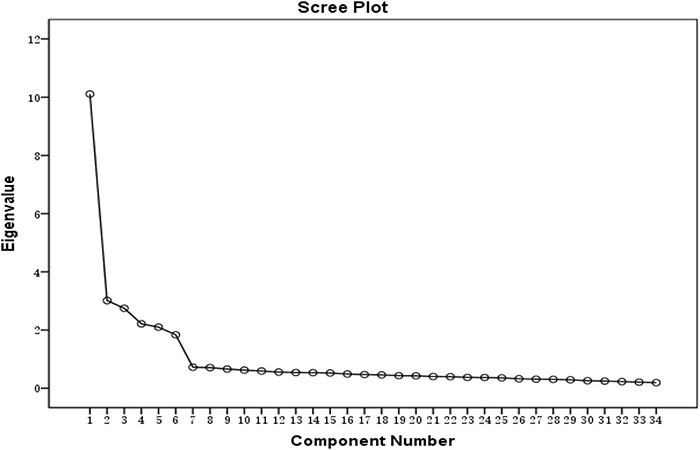
Scree plot.

It was found that the eigenvalues changed slowly after the sixth common factor. Therefore, it was more appropriate to select six common factors, and the following rotating component matrix was obtained (Figure 1).

The results are shown in Table 6. According to the meaning of the items in the scale and the rotating component matrix, if the load value was more significant than 0.5, it could be analysed as an essential item. The results showed that the load value of each item in each dimension was greater than 0.5. At the same time, the results of the rotating component matrix were consistent with the scale and dimensions divided by the research design. Therefore, the validity of the questionnaire was high, and the questionnaire was effective.

**TABLE 6:** Rotated component matrix.

	Component					
	1	2	3	4	5	6
A1 Being an entrepreneur implies more advantages than disadvantages to me.	0.722					
A2 A career as an entrepreneur is attractive to me.	0.718					
A3 If I had the opportunity and resources, I’d like to start a firm.	0.667					
A4 Being an entrepreneur would entail great satisfaction for me.	0.702					
A5 Among various options, I would rather be an entrepreneur.	0.728					
A6 I am ready to do anything to be an entrepreneur.	0.797					
A7 My life goal is to become an entrepreneur.	0.698					
A8 I will make every effort to start and run my own firm.	0.680					
A9 I have the intention to start a firm someday.	0.705					
B1 I believe that I can analyse long-term problems and find solutions.				0.725		
B2 Within the scope of my work, I believe I can help set goals/objectives.				0.758		
B3 I believe that I have contributed to the discussion of teamwork strategies.				0.875		
B4 I believe that I can contact people outside of the organisation (such as suppliers, customers, merchants) and discuss with them.				0.761		
B5 I believe that I can present information to a group of colleagues.				0.699		
C1 If I am involved in troubles at work, I can think of many ways to get rid of it.		0.759				
C2 At present, I am full of energy to complete my work goals.		0.793				
C3 At present, I think I am quite successful in my work.		0.686				
C4 At present, I am achieving the goals I set for myself.		0.891				
C5 I can find out many ways to achieve my current work goals.		0.755				
D1 It’s easy for me to recover from setbacks at work and move on.					0.725	
D2 I usually cope with pressure in my work easily.					0.710	
D3 I can solve most of the problems in my work.					0.732	
D4 I can complete the tasks by myself if they must be done.					0.888	
D5 In my current job, I reckon that I can handle many things simultaneously.					0.783	
G1 In my work, I usually expect the best results when I encounter uncertain things.			0.685			
G2 I always find out the positive side of things when I work.			0.783			
G3 I am optimistic about what will happen in the future of my work.			0.889			
G4 I always believe that “There will be a rainbow after every storm, so don’t be pessimistic.”			0.757			
G5 If something goes wrong, if I work wisely, it can be changed.			0.754			
H1 If I want to buy something, I always wait until I can afford it.						0.749
H2 Completing a problematic job can show your ability.						0.697
H3 s a middle-level manager of a company; you prefer to be the leader of a team that is not very good temporarily but with great potential.						0.733
H4 You will do all in your full strength to look for all chances to get the big client though there are many unexpected difficulties in the process.						0.730
H5 If your work is so stressful that you are uncomfortable to bearing with it, you will look for ways to relieve pressure and then continue to do a good job.						0.854

Extraction Method: Principal Component Analysis.						
Rotation Method: Varimax with Kaiser Normalisation.						
a. Rotation converged in 6 iterations.						

### Correlation Analysis

Through the pairwise correlation analysis of EI, self-efficacy, hope, flexibility, optimism, occupational delay of gratification, and PC, it was found that any two of them were in a significant correlation. There was a significant correlation between self-efficacy, hope, flexibility, optimism, occupational delay of gratification, and PC. Also, all of them had a significant correlation relationship with the EI. Additionally, it was suggested that self-efficacy, hope, resilience, optimism, occupational delay of gratification, and PC had significantly positive effects on EI (Table 7).

**TABLE 7:** Correlations.

	EI	Self-efficacy	Hope	Resilience	Optimism	Occupational delay of gratification	PC
EI	Pearson correlation	1	0.358**	0.322**	0.357**	0.313**	0.456**	0.483**
	Sig. (two-tailed)		0.000	0.000	0.000	0.000	0.000	0.000
	N	340	340	340	340	340	340	340
Self-efficacy	Pearson correlation	0.358**	1	0.377**	0.237**	0.280**	0.432**	0.675**
	Sig. (two-tailed)	0.000		0.000	0.000	0.000	0.000	0.000
	N	340	340	340	340	340	340	340
Hope	Pearson correlation	0.322**	0.377**	1	0.304**	0.329**	0.344**	0.726**
	Sig. (two-tailed)	0.000	0.000		0.000	0.000	0.000	0.000
	N	340	340	340	340	340	340	340
Resilience	Pearson correlation	0.357**	0.237**	0.304**	1	0.359**	0.319**	0.675**
	Sig. (two-tailed)	0.000	0.000	0.000		0.000	0.000	0.000
	N	340	340	340	340	340	340	340
Optimism	Pearson correlation	0.313**	0.280**	0.329**	0.359**	1	0.336**	0.712**
	Sig. (two-tailed)	0.000	0.000	0.000	0.000		0.000	0.000
	N	340	340	340	340	340	340	340
Occupational delay of gratification	Pearson correlation	0.456**	0.432**	0.344**	0.319**	0.336**	1	0.512**
	Sig. (two-tailed)	0.000	0.000	0.000	0.000	0.000		0.000
	N	340	340	340	340	340	340	340
PC	Pearson Correlation	0.483**	0.675**	0.726**	0.675**	0.712**	0.512**	1
	Sig. (two-tailed)	0.000	0.000	0.000	0.000	0.000	0.000	
	N	340	340	340	340	340	340	340

**Correlation is significant at the 0.01 level (two-tailed).

### Regression Analysis

According to Table 8, the adjusted *R*^2^ of the model was 0.240, the adjusted *R*^2^ was 0.231, and the *F*-value was 26.405. The significance probability was less than 0.05, which indicated that the model was to a good fitting degree. According to the coefficient table, the standardised regression coefficients of the independent variables of the model were 0.221, 0.129, 0.219, and 0.131, respectively. Additionally, the *t*-values were 4.212, 2.389, 4.168, and 2.457 individually, which passed the significance test (*p* < 0.05); according to the regression equation, self-efficacy, hope, resilience, and optimism showed a significantly positive impact on EI.

**TABLE 8:** The influence of psychological capital (PC) variables on entrepreneurial intention (EI).

Model	Unstandardised coefficients		Standardised coefficients	*t*	Sig.	*R* ^2^	Adjusted *R*^2^	*F*
						
	B	Std. Error	Beta					
(Constant)	1.569	0.189		8.306	0.000	0.240	0.231	26.405
Self- Efficacy	0.191	0.045	0.221	4.212	0.000			
Hope	0.107	0.045	0.129	2.389	0.017			
Resilience	0.191	0.046	0.219	4.168	0.000			
Hope	0.108	0.044	0.131	2.457	0.015			

*a. Dependent Variable: EI.*

According to the summary table of the model, the adjusted *R*^2^ of the model was 0.234, the adjusted *R*^2^ was 0.231, and the *F*-value was 103.087. The significance probability was less than 0.05, which indicated that the fitting degree of the model was of high quality. According to the coefficient table, the standardised regression coefficient of the model-independent variable was 10.153, and the *t*-value of the independent variable was 10.153, which passed the significance test (*p* < 0.05); and according to the regression equation, PC showed a significantly positive impact on the EI (Table 9).

**TABLE 9:** The influence of PC variables on EI.

Model	Unstandardised coefficients	Standardised coefficients	*t*	Sig.	*R* ^2^	Adjusted *R*^2^	*F*
						
	B	Std. Error	Beta					
(Constant)	1.598	0.188		8.504	0.000	0.234	0.231	103.087
PC	0.589	0.058	0.483	10.153	0.000			

*a. Dependent Variable: EI.*

According to Table 10, the adjusted *R*^2^ of the model is 0.208, the adjusted *R*^2^ is 0.206, and the *F*-value is 88.750. The significance probability is less than 0.05, indicating that the model has an excellent degree. According to the coefficient table, the standardised regression coefficient of the model-independent variable is 0.456, and the *t*-value of the independent variable was 9.421, passing the significance test (*p* < 0.05). According to the regression equation, career delay had a significantly positive impact on EI.

**TABLE 10:** The influence of occupational delay of gratification on EI.

Model	Unstandardised coefficients	Standardised coefficients	*t*	Sig.	*R* ^2^	Adjusted *R*^2^	*F*
						
	B	Std. Error	Beta					
(Constant)	2.225	0.138		16.179	0.000	0.208	0.206	88.750
Occupational Delay of Gratification	0.381	0.040	0.456	9.421	0.000			

*Dependent Variable: EI.*

According to the summary table of the model, the adjusted *R*^2^ of the model is 0.263, the adjusted *R*^2^ is 0.260, and the *F*-value is 120.400. The significance probability is less than 0.05, indicating that the model has an excellent fitting degree. According to the coefficient table, the standardised regression coefficient of the independent variable in the model was 0.512, and the *t*-value of the independent variable was 10.973, which passed the significance test (*p* < 0.05). Based on the regression equation, occupation delay had a significant positive impact on PC (Table 11).

**TABLE 11:** Analysis of the influence of occupational delay of gratification on PC.

Model	Unstandardised coefficients	Standardised coefficients	*t*	Sig.	*R* ^2^	Adjusted *R*^2^	*F*
						
	B	Std. Error	Beta					
(Constant)	2.026	0.109		18.599	0.000	0.263	0.260	120.400
Occupational Delay of Gratification	0.352	0.032	0.512	10.973	0.000			

*Dependent Variable: PC.*

### Mediating Effect Test

By using the SPSS compiled by Hayes (2012), the results showed that in the model with the occupational delay of gratification as an independent variable and psychological resilience as a dependent variable, *R* was 0.263 and the *F*-value was 120.400, indicating that the model matching degree was in good quality, and the independent variable coefficient was positive. As shown by the significance test, the independent variable had a significant positive impact on the dependent variable. In the model with the occupational delay of gratification and PC as an independent variable and EI as a dependent variable, the *R* was 0.293, and the *F*-value was 0.293, which meant a good matching degree of the model. Meanwhile, the independent variable coefficient was positive, passing the significance test. As a result, it implied that the independent variable had a significant positive impact on the dependent variable. In the model with the occupational delay of gratification as the independent variable and EI as the dependent variable, *R* was 0.208, *F*-value was 88.751, showing that the model matching degree was accurate. In this process, the independent variable coefficient was positive, which passed the significance test, implying that the independent variable had a significant positive impact on the dependent variable (Table 12).

**TABLE 12:** Mediating effect test.

	PC			EI			EI		
	B	*t*	*p*	B	*t*	*p*	B	*t*	*p*
(Constant)	2.026	18.599	0.000	1.389	7.503	0.000	2.225	16.179	0.000
Occupational delay of gratification	0.352	10.973	0.000	0.236	5.294	0.000	0.381	9.421	0.000
PC				0.412	6.347	0.000			
*R* ^2^	0.263			0.293			0.208		
F	120.400			69.677			88.751		

According to Table 13, the upper and lower limits of the 95% confidence interval of PC did not contain 0 value. It means that there was a mediating effect. In detail, the occupational delay of gratification could not only predict EI directly but also predict EI *via* the mediating role of PC.

**TABLE 13:** The bootstrap mediating effect test.

	Effect	Boot SE	Boot LLCI	Boot ULCI
The mediating effect of PC	0.145	0.028	0.094	0.204

## Discussion

As mentioned in the literature review, EA and EI were in a strong relationship which was the foundation of PC (Gough, 2019). Entrepreneurial attitude could affect an individual’s behaviour through intention. So the research was conducted by offering a questionnaire to measure students’ EI in IBSS *via* the first nine items. All the objectives selected met the requirements of maintaining a strong EI. Although we did not include those potential students with strong EI by the basic questions, such as whether their relatives or friends had their own business, the research excluded the external factor variables of the inter-generation effect and the crowed following effect. It was imperative to ensure their intention so that the mediator factors and the independent variables were able to be included in the model. Then, a strong relationship between EI and PC has been reported in the literature. Mahfud (2019) mentioned that the PC as the mediator could influence students’ EA on EI to start new businesses in their previous research. This kind of relationship was also identified among students in IBSS. Those students with good PC, especially those with high resilience, were more likely to start a new business. Besides that, several reports have shown that it was efficient to foster students to be entrepreneurs by improving their PC skills (Ephrem et al., 2019). It was also in the same situation when taking students in IBSS as the sample. Students can be affected by adjusting the factors of PC. Those tests could identify that the students in IBSS fitted the relationship between EI and PC. Consequently, this research successfully demonstrated the hypothesis that PC had a positive prediction of students’ EI in IBSS directly.

In the research of Tolentino (2014), career adaptability as the form of occupational delay of gratification was identified to be a vital major in predicting self-efficacy and EI positively. It was also demonstrated among students in IBSS. However, one interesting finding is that not only self-efficacy but also other kinds of elements can also be affected by the occupational delay of gratification positively. According to the test of the mediator, it was found that there was a significant correlation between independent variables. As a result, students in IBSS can be affected and directed to improve their cognition of self-efficacy, hope, capacity to maintain optimism, and resilience by cultivating the occupational delay of gratification. Most of the research focused on employed people but not students. It is also why students were the objective to identify the relationship between the occupational delay of gratification and the PC in this research. Then, it could also be found that the second hypothesis was correct. In IBSS, students’ occupational delay of gratification could impinge on their PC positively.

Another finding was that occupational delay of gratification could affect students’ EI *via* PC, which meant that a high level of occupational delay of gratification could stimulate students in IBSS to obtain the EI. In reviewing the literature, no data were found on the association between the occupational delay of gratification and EI. In this research, students were demonstrated to obtain the EI by enhancing the occupational delay of gratification. It can be deduced that a high level of gratification is a trait of students who intend to be an entrepreneur in the future. Also, it proved the third hypothesis as follows: occupational delay of gratification positively predicts students’ EI. As a result, PC can be determined to be the intermediary variable in the relationship between the occupational delay of gratification and EI among students in IBSS.

All the tests conducted proved those hypotheses, but the results should be considered regarding the study’s limitations. Participants were selected at the beginning of the research to ensure all the samples started up new businesses. However, some samples planning or deciding to join entrepreneurial activities were not included. It means that some external factors were not explored, which was also able to impact students’ EI. It was not clear whether the external factors would change the current relationship. Besides that, samples were from IBSS, who were business students preferring to join entrepreneurship and business-related activities. In the following research, the relationship among those three variables is expected to be demonstrated in other target groups like students majoring in humanities and natural science. In other words, the relationship could not be used commonly. Therefore, an extensive scope of samples is required in future research, and the external factors should be taken into account based on the current relationship among those three variables.

## Data Availability Statement

The original contributions presented in this study are included in the article/supplementary material, further inquiries can be directed to the corresponding authors.

## Author Contributions

BN: conceptualization, methodology, software, and writing – original draft preparation. NA: validation, formal analysis, investigation, resources, supervision, funding acquisition, and supervision. CZ: data curation, writing – review and editing, and visualization. YH: writing – original draft Preparation and data collection. All authors have read and agreed to the published version of the manuscript.

## Conflict of Interest

The authors declare that the research was conducted in the absence of any commercial or financial relationships that could be construed as a potential conflict of interest.

## Publisher’s Note

All claims expressed in this article are solely those of the authors and do not necessarily represent those of their affiliated organizations, or those of the publisher, the editors and the reviewers. Any product that may be evaluated in this article, or claim that may be made by its manufacturer, is not guaranteed or endorsed by the publisher.

## Appendix

**Questionnaire**

Please complete the 5 questions ahead before starting the following questions.

**A. (Basic Information)**

1. Do you already have your own business?

2. Does someone in your family has their own business?

3. Do any of your friends have their own business?

4. Your age?

5. Your gender?

Please indicate your level of agreement with the following statements from 1 (total disagreement) to 5 (total agreement).

**B. (Entrepreneurial Intention)**

1. Being an entrepreneur implies more advantages than disadvantages to me.

2. A career as an entrepreneur is attractive to me.

3. If I had the opportunity and resources, I’d like to start a firm.

4. Being an entrepreneur would entail great satisfaction for me.

5. Among various options, I would rather be an entrepreneur.

6. I am ready to do anything to be an entrepreneur.

7. My life goal is to become an entrepreneur.

8. I will make every effort to start and run my own firm.

9. I have the intention to start a firm someday.

**C. (Psychological Capital)**

**a. (Self-efficacy)**

10. I believe that I can analyse long-term problems and find solutions.

11. Within the scope of my work, I believe I can help set goals/objectives.

12. I believe that I have contributed to the discussion of teamwork strategies.

13. I believe that I can contact people outside of the organisation (such as suppliers, customers, merchants) and discuss with them.

14. I believe that I can present information to a group of colleagues.

**b. (Hope)**

15. If I am involved in troubles at work, I can think of many ways to get rid of it.

16. At present, I am full of energy to complete my work goals.

17. At present, I think I am quite successful in my work.

18. At present, I am achieving the goals I set for myself.

19. I can find out many ways to achieve my current work goals.

**c. (Resilience)**

20. It’s easy for me to recover from setbacks at work and move on.

21. I usually cope with pressure in my work easily.

22. I can solve most of the problems in my work.

23. I can complete the tasks by myself if they must to be done.

24. In my current job, I reckon that I can handle many things simultaneously.

**d. (Optimism)**

25. In my work, I usually expect the best results when I encounter uncertain things.

26. I always find out the positive side of things when I work.

27. I am optimistic about what will happen in the future of my work.

28. I always believe that “There will be a rainbow after every storm, so don’t be pessimistic.”

29. If something goes wrong, if I work wisely, it can be changed.

**D. (Occupational Delay of Gratification)**

30. If I want to buy something, I always wait until I can afford it.

31. Completing a difficult job can show your ability.

32. As a middle-level manager of a company, you prefer to be the leader of a team that is not very good temporarily but with great potentiality.

33. Your will do all in your full strength to look for all chances to get the big client though there are many unexpected difficulties in the process.

34. If your work is so stressful that you are uncomfortable to bear with it, you will look for ways to relieve pressure and then continue to do a good job.
